# Genetic influence of CYP2D6 on pharmacokinetics and acute subjective effects of LSD in a pooled analysis

**DOI:** 10.1038/s41598-021-90343-y

**Published:** 2021-05-25

**Authors:** Patrick Vizeli, Isabelle Straumann, Friederike Holze, Yasmin Schmid, Patrick C. Dolder, Matthias E. Liechti

**Affiliations:** Division of Clinical Pharmacology and Toxicology, Department of Biomedicine and Department of Clinical Research, University Hospital Basel, Schanzenstrasse 55, 4056 Basel, Switzerland

**Keywords:** Pharmacogenetics, Pharmacokinetics

## Abstract

Lysergic acid diethylamide (LSD) is a classic psychedelic substance that is used recreationally and investigated in psychiatric research. There are no pharmacogenetic studies on LSD. In vitro metabolic studies indicate that several cytochrome P450 (CYP) isoforms (e.g., CYP2D6, CYP1A2, and CYP2C9) are involved in LSD metabolism, but in vivo data are scarce. The present study examined the influence of genetic polymorphisms of CYP genes on the pharmacokinetics and acute effects of LSD in healthy subjects. We identified common genetic variants of CYPs (CYP2D6, CYP1A2, CYP2C9, CYP2C19, and CYP2B6) in 81 healthy subjects who were pooled from four randomized, placebo-controlled, double-blind Phase 1 studies. We found that genetically determined CYP2D6 functionality significantly influenced the pharmacokinetics of LSD. Individuals with no functional CYP2D6 (i.e., poor metabolizers) had longer LSD half-lives and approximately 75% higher parent drug and main metabolite 2-oxo-3-hydroxy LSD area-under-the-curve blood plasma concentrations compared with carriers of functional CYP2D6. Non-functional CYP2D6 metabolizers also exhibited greater alterations of mind and longer subjective effect durations in response to LSD compared with functional CYP2D6 metabolizers. No effect on the pharmacokinetics or acute effects of LSD were observed with other CYPs. These findings indicate that genetic polymorphisms of CYP2D6 significantly influence the pharmacokinetic and subjective effects of LSD. Given the potential therapeutic use of psychedelics, including LSD, the role of pharmacogenetic tests prior to LSD-assisted psychotherapy needs to be further investigated.

## Introduction

Lysergic acid diethylamide (LSD) is a classic psychedelic. After early psychiatric research, recreational use, and prohibition, LSD was rediscovered by modern psychiatric research and may be useful for LSD-assisted psychotherapy^1–6^. However, despite its increasing use, the metabolism of LSD is not fully understood. Two recent in vitro studies reported the involvement of cytochrome P450 (CYP) enzymes in the metabolism of LSD^7,8^. One study of human liver microsomes showed that CYP2D6, CYP3A4, and CYP2E1 contribute to the *N*-demethylation of LSD to 6-nor-LSD (nor-LSD), whereas CYP2C9, CYP1A2, CYP2E1, and CYP3A4 participate in the formation of LSD’s main metabolite 2-oxo-3-hydroxy-LSD (O-H-LSD)^8^. Another study of human liver S9 fractions reported that CYP2C19 and CYP3A4 were involved in the formation of nor-LSD, and CYP1A2 and CYP3A4 contributed to the hydroxylation of LSD^7^. Some CYP enzymes (e.g., CYP2D6, CYP1A2, CYP2C9, and CYP2C19) have common functional genetic polymorphisms that result in different phenotypes^9–14^. CYP2D6 is associated with several phenotypes, ranging from poor metabolizers (PMs; 5–10% in Caucasians) to ultra-rapid metabolizers (UMs; 3–5% in Caucasians), with different underlying genotypes^11^. CYP2D6 genotype has previously been shown to influence the pharmacokinetics of 3,4-methylenedioxymethamphetamine (MDMA)^15,16^, a substance that is also used for substance-assisted psychotherapy^6^. Genetic variants of LSD-metabolizing CYPs, especially CYP2D6^8^, may also influence the pharmacokinetics of LSD. Additionally, it has been shown that the acute effects of LSD are dose-dependent and closely linked to the plasma concentration–time curve of LSD within an individual. Thus, the acute pharmacodynamic effects of LSD may also be critically influenced by CYP pharmacogenetics^17–19^.

The present study investigated the influence of prominent genetic polymorphisms of several CYPs involved in the metabolism of LSD (i.e., CYP2D6, CYP1A2, CYP2C9, CYP2C19, and CYP2B6) on pharmacokinetic parameters of LSD and its acute subjective effects. Based on in vitro studies with LSD, we hypothesized that CYP2D6 PMs would exhibit higher LSD concentrations and acute effects compared with individuals with functional CYP2D6. The quality and extent of subjective effects of psychedelics are particularly interesting because more intense and more positive acute psychedelic effects are thought to predict long-term therapeutic outcome in patients who receive psychedelic-assisted therapy^20–22^ and also positive long-term effects in healthy subjects^23,24^.

## Methods

### Study design

This was a pooled secondary analysis of four Phase 1 studies that each used a randomized, double-blind, placebo-controlled, crossover design and were conducted in the same laboratory^17,25–27^. The studies were all registered at ClinicalTrials.gov (Study 1: NCT01878942; Study 2: NCT02308969; Study 3: NCT03019822; Study 4: NCT03321136). The studies included a total of 84 healthy subjects. Study 1^25^ and Study 4^17^ each included 16 subjects. Study 2 included 24 subjects^26^. Study 3 included 29 subjects^27^. In Study 1, each subject received a single dose of 200 µg LSD or placebo. In Studies 2 and 3, each subject received a single dose of 100 µg LSD or placebo. In Study 4, each subject received 25, 50, 100, and 200, and 200 µg LSD + 40 mg ketanserin (a serotonin [5-hydroxytryptamine, 5-HT] 2A receptor antagonist). For this pooled analysis, we used mean data of the four LSD doses that were used within the same subject in Study 4. The 200 µg LSD + 40 mg ketanserin condition was used for the pharmacokinetic analysis but not for the analysis of the effect of LSD. All of the studies were approved by the local ethics committee (Ethikkommission Nordwest- und Zentralschweiz) and were conducted in accordance with the Declaration of Helsinki. The use of LSD was authorized by the Swiss Federal Office for Public Health (Bundesamt für Gesundheit), Bern, Switzerland. Written informed consent was obtained from all of the subjects. All of the subjects were paid for their participation. The washout periods between doses were 7 days for Studies 1 and 2 and 10 days for Studies 3 and 4. Test sessions were conducted in a quiet hospital research ward with no more than one research subject present per session. The subjects were under constant supervision while they experienced acute drug effects. The subjects comfortably reclined in hospital beds and were mostly listening to music and not engaging in physical activities. LSD was given after a standardized small breakfast in the morning. A detailed overview of the four studies is shown in Supplementary Table S1.

### Subjects

A total of 85 healthy subjects of European descent, 25–60 years old (mean ± SD = 30 ± 8 years), were recruited from the University of Basel campus or word-of-mouth advertising and participated in the study. Two participants withdrew from the study, one before the first test session and one before the final LSD session. Two participants did not give consent for genotyping. These four participants were excluded from the final dataset, resulting in a final total of 81 subjects (41 women). The subjects’ mean ± SD body weight was 70 ± 12 kg (range: 50–98 kg). Participants who were younger than 25 years old were excluded from participating in the study because of the higher incidence of psychotic disorders in this age group and because younger ages have been associated with more anxious reactions to hallucinogens^28^. The exclusion criteria of all the analyzed parent studies included a history of psychiatric disorders, physical illness, tobacco smoking (> 10 cigarettes/day), use of any medication that may interfere with the effects of the study medications, a lifetime history of illicit drug use more than 10 times (with the exception of past cannabis use), illicit drug use within the past 2 months, and illicit drug use during the study, determined by urine tests that were conducted before the test sessions. Twenty-two subjects had prior hallucinogenic drug experiences, of which 16 subjects had previously used LSD (1–3 times), five subjects had previously used psilocybin (1–3 times), and one subject had previously used dimethyltryptamine (4 times), mescaline (1 time), and salvia divinorum (3 times).

### Study drug

LSD base (Lipomed AG, Arlesheim, Switzerland) was prepared to be taken orally as gelatin capsules^25,26^ in Studies 1 and 2 and as a drinking solution in 96% ethanol in Studies 3 and 4^17,27^. The doses that were used in each study are shown in Supplementary Table S1. Content uniformity and long-term stability data were available for the doses that were used in Studies 3 and 4^17,19,27^. The exact actual mean doses of LSD base administered are shown in Supplementary Table S1. The planned mean doses that were used in Studies 1 and 2 were later detected to be lower, and the actual doses that were used were estimated based on comparisons of area-under-the-curve (AUC) values from Studies 1 and 2 with AUC values from Studies 3 and 4^19^. The LSD doses of the analyzed studies were within the range usually used for therapeutic sessions and were not adjusted for body weight or sex^6,29^.

### Pharmacokinetic analyses

**Figure 1:**
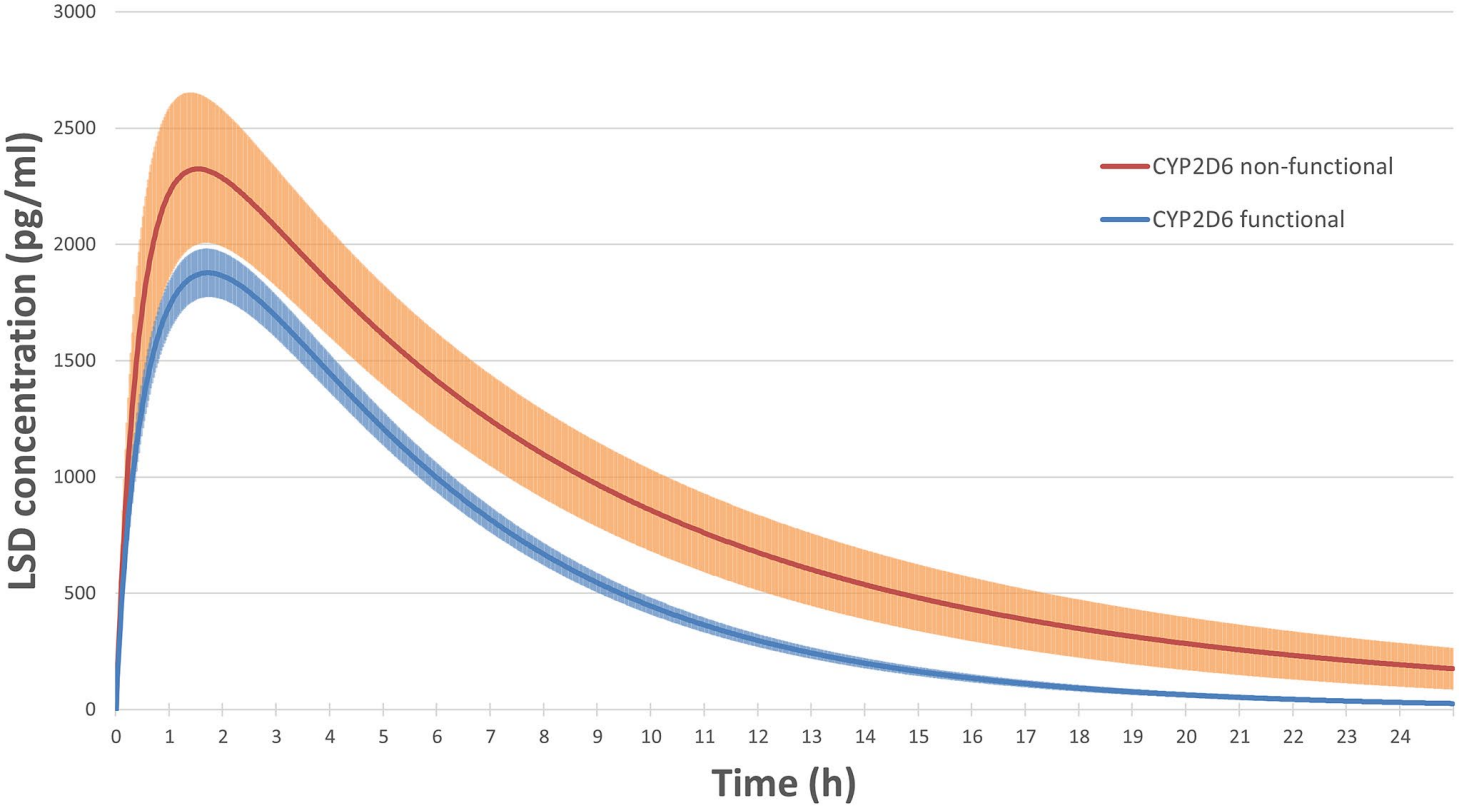
Modeled plasma LSD concentration–time curves over 24 h after LSD administration in subjects with genetically determined non-functional (red) or functional (blue) CYP2D6 enzymes. The shaded area marks the standard error of the mean. Non-functional (*n* = 7) and functional (*n* = 74) CYP2D6 subjects received a dose of 100 ± 30 µg LSD and 98 ± 35 µg LSD (mean ± SD), respectively. Both the half-life and AUC values significantly increased in subjects with non-functional CYP2D6 compared with functional CYP2D6.

Pharmacokinetic parameters were calculated using non-compartmental analysis in Phoenix WinNonlin 6.4 (Certara, Princeton, NJ, USA). Peak effect (E_max_) values were obtained directly from the observed data. AUC and area under the time-effect curve AUEC values were calculated using the linear-log trapezoidal method. AUC values were calculated up to the last measured concentration in all studies (AUC_10_) and extrapolated to infinity (AUC_∞_). Additionally, a one-compartment model with first-order input, first-order elimination, and no lag time was used in Phoenix WinNonlin 6.4. to compare the pharmacokinetics of LSD among functional and non-functional CYP2D6 groups and to illustrate the LSD concentrations over time (Fig. 1) after a dose of 100 µg LSD base. This analysis included the data from all 81 subjects. For Study 4, only the 100 µg dose was included.

### Physiological effects

Blood pressure, heart rate, and body temperature were assessed before and repeatedly after LSD or placebo administration^17,25–27^. Mean arterial pressure was calculated as *diastolic blood pressure* + *(systolic blood pressure – diastolic blood pressure)/3*. The rate pressure product was calculated as *systolic blood pressure* × *heart rate.* Core (tympanic) temperature was measured using a Genius 2 ear thermometer (Tyco Healthcare Group LP, Watertown, NY, USA).

### Subjective effects

Visual Analog Scales (VASs) were presented as 100 mm horizontal lines (0–100%), marked from “not at all” on the left to “extremely” on the right. The VASs were applied before and repeatedly after LSD or placebo administration^17,25–27^. The onset, offset, and duration of the subjective response were determined using the VAS “any drug effect”-time curve, with 10% of the individual maximal response as the threshold, in Phoenix WinNonlin.

The 5 Dimensions of Altered States of Consciousness (5D-ASC) scale^30,31^ was administered at the end of the acute drug effects to retrospectively rate peak drug responses. The main subscales that describe alterations of consciousness are Oceanic Boundlessness (OB), Anxious Ego Dissolution (AED), and Visionary Restructuralization (VR).

### Genotyping

Genomic DNA was extracted from whole blood using the QIAamp DNA Blood Mini Kit (Qiagen, Hombrechtikon, Switzerland) and an automated QIAcube system. Single-nucleotide polymorphism (SNP) genotyping was performed using commercial TaqMan SNP genotyping assays (LuBio Science, Lucerne, Switzerland). We were looking at the most common SNPs and haplotypes for the selected CYPs within the population of European ancestry. Nevertheless, we also covered SNPs with a minor allele frequency (MAF) < 1% to catch or rule out mutations with substantial impact (i.e. CYP2D6*3 or CYP2C19*4). We did run uncertain assays twice or more if needed, with duplicates and controls with known genotypes for confirmation. In addition, two experts reviewed the data independently. We assayed the following SNPs and respective alleles: CYP1A2*1F (rs762551, assay: C___8881221_40), CYP2B6 (rs3745274, assay: C___7817765_60), CYP2C9*2 (rs1799853, assay: C__25625805_10), CYP2C9*3 (rs1057910, assay: C__27104892_10), CYP2C19*2 (rs4244285, assay: C__25986767_70), CYP2C19*4 (rs28399504, assay: C__30634136_10), CYP2C19*17 (rs12248560, assay: C____469857_10), CYP2D6*3 (rs35742686, assay: C_32407232_50), CYP2D6*4 (rs3892097, assay: C_27102431_D0; rs1065852, assay: C_11484460_40), CYP2D6*6 (rs5030655, assay: C_32407243_20), CYP2D6*9 (rs5030656, assay: C__32407229_60), CYP2D6*10 (rs1065852, assay: C_11484460_40), CYP2D6*17 (rs28371706, assay: C_2222771_A0; rs16947, assay: C_27102425_10), CYP2D6*29 (rs59421388, assay: C_3486113_20), and CYP2D6*41 (rs28371725, assay: C_34816116_20; rs16947, assay: C_27102425_10). CYP2D6 gene deletion (allele *5) and duplication/multiplication (allele *xN) were determined using a TaqMan Copy Number Assay (Hs04502391_cn). Activity scores for CYP2D6 were assigned according to established guidelines^10,12,32–34^. To see a distinct effect of CYP2D6 functionality on the pharmacokinetic and pharmacodynamic effects of LSD, we classified the subjects as non-functional CYP2D6 (PMs; activity score = 0) and functional CYP2D6 (activity score > 0). The activity score for CYP2C9 was generated using the relative metabolic activity of warfarin^35,36^. Genetically determined CYP1A2 activity inducibility was combined with the smoking status of the subject (> 5 cigarettes per day = smoker; rs762551 AA = inducible)^9,15^. Predicted CYP2C19 intermediate metabolizers (IMs) included CYP2C19*1/*2 and CYP2C19*2/*17, extensive metabolizers (EMs) included CYP2C19*1/*1, and UMs included both CYP2C19*17/*17 and CYP2C19*1/*17^10^. No CYP2C19 PM was identified within the sample. For CYP2B6, we determined the reduced-activity rs3745274 SNP (516G>T, CYP2B6*6 or CYP2B6*9, assay: C_7817765_60). Allele frequencies for the classification of CYP2D6 and CYP2C9 are shown in Supplementary Tables S2 and S3, respectively. All of the tested SNP frequencies were comparable to the Allele Frequency Aggregator Project databank and are listed in Supplementary Table S4^37^.

### Statistical analysis

All of the data were analyzed using the R language and environment for statistical computing^38^. To test for genotype effects, the pharmacokinetic parameters or effects of LSD (Δ LSD-placebo) were compared using one-way analysis of variance (ANOVA), with genotype as the between-group factor. The data are presented as actual nominal values and z-scores per study because the nominal values may be biased by a possible unequal distribution of genotypes across studies using different doses. The statistics were not corrected for sex or body weight because we found no correlations between sex/bodyweight and exposure to the drug (LSD AUC_∞_; Supplementary Fig. S1). As shown in Supplementary Fig. S1, an outlying individual was identified as non-functional CYP2D6. To minimize the effect of outliers and associated non-normal data distributions on the parametric statistics, we confirmed the results for the influence of CYP2D6 functionality on the pharmacokinetics and effects of LSD with nonparametric statistics (Wilcoxon signed-rank test and Kruskal–Wallis test). The level of significance was set at *p* < 0.05. Values of *p* in the pharmacokinetic analysis were not corrected for multiple testing because hypotheses for the influence of certain enzyme activities (i.e., CYP2D6) were made a priori.

## Results

LSD produced significant acute subjective effects on all scales and moderately increased blood pressure, heart rate, and body temperature compared with placebo (Supplementary Table S5). Sex and body weight did not significantly alter the pharmacokinetics or acute effects of LSD (Supplementary Fig. S1).

### Effects of CYP genotype on LSD pharmacokinetics and acute effects

CYP2D6 function significantly influenced the pharmacokinetics and acute effects of LSD (Table 1, Fig. 1). Specifically, subjects who were genetically classified as CYP2D6 PMs (non-functional) exhibited higher plasma LSD exposure (Fig. 1), reflected by significantly larger AUC_∞_ and AUC_10_ values compared with functional CYP2D6 carriers (Table 1). CYP2D6 PMs also had longer T_1/2_ values, consistent with slower metabolism compared with functional CYP2D6 subjects (Table 1), whereas the C_max_ of LSD was not significantly affected. Furthermore, O-H-LSD AUC_∞_ values were larger in CYP2D6 PMs compared with functional CYP2D6 subjects (Table 1), in parallel with the effects on LSD concentrations and indicating that the conversion to O-H-LSD occurred independently of CYP2D6. Compartmental modeling for 100 µg LSD administration showed LSD AUC_∞_ and C_max_ values for CYP2D6 PMs *vs*. functional CYP2D6 subjects of (mean ± SD) 24,169 ± 13,112 *vs*. 13,819 ± 6281 pg/ml*h (*F*_1,79_ = 13.8, *p* < 0.001) and 2369 ± 891 *vs*. 2061 ± 999 pg/ml (*F*_1,79_ = 0.62, *p* = 0.43), respectively (Fig. 1). Lower CYP2D6 activity was also associated with significantly higher exposure to LSD when analyzed across all CYP2D6 genotype activity score groups (Supplementary Table S6).

**Table 1:** Effects of genetically determined function of cytochromes P450 2D6 on the pharmacokinetics and response to LSD [mean ± SD (N)] with non-corrected statistics (non- and parametric) of *the nominal values* and z-scores (per study).

	Genotype CYP2D6 functionality		F	p value	η^2^	W	p value^a^
	Non-functional	Functional					
N	7	74					
Female, N [%]	3 [43]	38 [51]					
CYP 2D6 PMs, N [%]	7 [100]	0 [0]					
Dose 1	107 ± 31	100 ± 36					
Dose 2	98 ± 30	97 ± 35					
Bodyweight, kg	71.0 ± 15	69.9 ± 12	0.05	NS	0.00	255	NS
Dose per bodyweight, μg/kg	1.6 ± 0.5	1.5 ± 0.6	0.19	NS	0.00	225	NS
**Pharmacokinetic parameters**							
LSD T_1/2_, h	*7.5* ± *6.2*	*4.1* ± *1.5*	*14.94*	< 0.001***	*0.16*	*134*	0.036*
	1.1 ± 1.6	− 0.078 ± 0.85	10.64	0.002**	0.12	152	0.073
LSD T_max_, h	*1.8* ± *0.8*	*1.7* ± *0.7*	*0.17*	*NS*	*0.00*	*240*	*NS*
	0.27 ± 1.3	− 0.029 ± 0.94	0.58	NS	0.01	237	NS
LSD C_max_, pg/mL	*2517* ± *878*	*2207* ± *1068*	*0.55*	*NS*	*0.01*	*186*	*NS*
	0.4 ± 1.0	− 0.032 ± 0.98	1.25	NS	0.02	176	NS
LSD AUC_inf_, h × pg/mL	*27,389* ± *18,859*	*14,673* ± *7349*	*13.44*	< 0.001***	*0.15*	*117*	0.017*
	1.1 ± 1.4	− 0.089 ± 0.88	10.55	0.002**	0.12	110	0.013*
LSD AUC_10h_, h × pg/mL	*16,730* ± *6921*	*11,753* ± *5139*	*5.65*	0.020*	*0.07*	*137*	0.041*
	0.85 ± 1.2	− 0.068 ± 0.93	5.95	0.017*	0.07	134	0.036*
O-H-LSD T_1/2_, h	*16* ± *10 (5)*	*9.3* ± *11 (47)*	*2.10*	*NS*	*0.04*	*39*	0.012*
	0.89 ± 1.6 (5)	− 0.095 ± 0.87 (47)	4.94	0.031*	0.09	53	0.045*
O-H-LSD T_max_, h	*6.2* ± *1.8 (6)*	*4.5* ± *1.6 (53)*	*5.67*	0.021*	*0.09*	*83*	*0.056*
	0.97 ± 1.1 (6)	− 0.11 ± 0.92 (53)	7.21	0.009**	0.11	71	0.027*
O-H-LSD C_max_, pg/mL	*134* ± *40 (6)*	*105* ± *54 (53)*	*1.58*	*NS*	*0.03*	*107*	*NS*
	0.33 ± 1.2 (6)	− 0.037 ± 0.97 (53)	0.74	NS	0.01	118	NS
O-H-LSD AUC_inf_, h × pg/mL	*3068* ± *1000 (5)*	*1630* ± *1464 (47)*	*4.57*	0.037*	*0.08*	*34*	0.007**
	1.6 ± 0.9 (5)	− 0.17 ± 0.84 (47)	18.92	< 0.001***	0.28	14	< 0.001***
O-H-LSD AUC_10h_, h × pg/mL	*1010* ± *342 (6)*	*747* ± *412 (53)*	*2.26*	*NS*	*0.04*	*102*	*NS*
	0.54 ± 1.5 (6)	− 0.061 ± 0.9 (53)	2.06	NS	0.04	108	NS
**Visual analog scale rating ΔE**_**max**_							
Subjective effect duration, h	*11.8* ± *6.2*	*8.9* ± *3.3*	*4.33*	0.041*	*0.05*	*195*	*NS*
	0.64 ± 1.33	− 0.06 ± 0.93	3.40	0.069	0.04	177	NS
Any drug effect	*79* ± *19*	*83* ± *19*	*0.37*	*NS*	*0.01*	*295*	*NS*
	0.18 ± 0.74	− 0.018 ± 1	0.27	NS	0.00	244	NS
Good drug effect	*74* ± *20*	*81* ± *21*	*0.71*	*NS*	*0.01*	*322*	*NS*
	0.023 ± 0.59	− 0.002 ± 1.0	0.00	NS	0.00	300	NS
Bad drug effect	*19* ± *22*	*26* ± *27*	*0.44*	*NS*	*0.01*	*280*	*NS*
	− 0.086 ± 0.9	0.008 ± 0.99	0.06	NS	0.00	264	NS
**Vital signs parameters ΔE**_**max**_							
Mean arterial pressure, mmHg	*9.5* ± *6.6*	*7.0* ± *9.2*	*0.47*	*NS*	*0.01*	*220*	*NS*
	0.41 ± 0.77	− 0.039 ± 0.99	1.37	NS	0.02	181	NS
Heart rate, bpm	*14* ± *8*	*12* ± *12*	*0.23*	*NS*	*0.00*	*215*	*NS*
	0.34 ± 0.47	− 0.032 ± 1	0.93	NS	0.01	185	NS
Body temperature, °C	*0.2* ± *0.31*	*0.37* ± *0.37*	*1.42*	*NS*	*0.02*	*381*	0.042*
	− 0.27 ± 0.94	0.025 ± 0.99	0.57	NS	0.01	322	NS
Rate pressure product, mmHg × bpm	*3102* ± *1582*	*2383* ± *2169*	*0.73*	*NS*	*0.01*	*181*	*NS*
	0.44 ± 0.41	− 0.042 ± 1	1.58	NS	0.02	154	0.079
**Dimensional altered state of consciousness, %**							
5D-ASC total	*42* ± *22*	*31* ± *16*	*2.65*	*NS*	*0.03*	*183*	*NS*
	1.1 ± 0.8	− 0.11 ± 0.93	11.48	0.001**	0.13	78	0.002**
Oceanic BOUNDLESSNESS	*50* ± *30*	*40* ± *24*	*0.95*	*NS*	*0.01*	*212*	*NS*
	0.69 ± 0.89	− 0.065 ± 0.97	3.88	0.052	0.05	145	0.056
Anxious ego dissolution	*37* ± *30*	*19* ± *16*	*6.92*	0.010*	*0.08*	*142*	*0.050*
	1.2 ± 1.2	− 0.12 ± 0.89	13.65	< 0.001***	0.15	90	0.005**
Visionary restructuralization	*55* ± *16*	*47* ± *23*	*0.76*	*NS*	*0.01*	*212*	*NS*
	0.84 ± 0.65	− 0.079 ± 0.97	5.89	0.017*	0.07	112	0.014*
Auditory alteration	*18* ± *21*	*14* ± *16*	*0.45*	*NS*	*0.01*	*238*	*NS*
	0.7 ± 1.5	− 0.066 ± 0.9	4.08	0.047*	0.05	186	NS
Vigilance reduction	*48* ± *30*	*33* ± *22*	*2.74*	*NS*	*0.03*	*180*	*NS*
	0.83 ± 0.81	− 0.078 ± 0.96	5.76	0.019*	0.07	127	0.027*
Experiance of unity	*54* ± *38*	*40* ± *27*	*1.60*	*NS*	*0.02*	*200*	*NS*
	0.67 ± 1.1	− 0.063 ± 0.96	3.69	0.058	0.05	151	0.071
Spiritual experience	*30* ± *35*	*21* ± *23*	*1.02*	*NS*	*0.01*	*222*	*NS*
	0.39 ± 0.99	− 0.037 ± 0.98	1.21	NS	0.02	190	NS
Blissful state	*43* ± *29*	*42* ± *33*	*0.01*	*NS*	*0.00*	*237*	*NS*
	0.17 ± 0.59	− 0.016 ± 1	0.23	NS	0.00	210	NS
Insightfulness	*38* ± *30*	*30* ± *30*	*0.52*	*NS*	*0.01*	*183*	*NS*
	0.48 ± 1.0	− 0.046 ± 0.97	1.89	NS	0.02	162	NS
Disembodiment	*57* ± *36*	*37* ± *31*	*2.60*	*NS*	*0.03*	*167*	*NS*
	0.98 ± 0.64	− 0.093 ± 0.96	8.37	0.005**	0.10	94	0.006**
Impaired control and cognition	*50* ± *28*	*32* ± *24*	*3.38*	*0.070*	*0.04*	*160*	*0.098*
	1.1 ± 1	− 0.11 ± 0.91	11.86	0.001**	0.13	101	0.008**
Anxiety	*27* ± *35*	*9* ± *15*	*6.98*	0.010*	*0.08*	*124*	0.023*
	1.0 ± 1.7	− 0.099 ± 0.84	9.67	0.003**	0.11	134	0.036*
Complex imagery	*73* ± *23*	*48* ± *33*	*3.88*	*0.052*	*0.05*	*140*	0.046*
	1.0 ± 0.7	− 0.099 ± 0.95	9.72	0.003**	0.11	94	0.006**
Elementary imagery	*71* ± *26*	*60* ± *32*	*0.72*	*NS*	*0.01*	*206*	*NS*
	0.71 ± 0.47	− 0.067 ± 0.99	4.17	0.044*	0.05	139	0.044*
Audio visual synesthesiae	*64* ± *26*	*65* ± *37*	*0.01*	*NS*	*0.00*	*266*	*NS*
	0.4 ± 0.79	− 0.037 ± 0.99	1.25	NS	0.02	199	NS
Changed meaning of percepts	*50* ± *27*	*41* ± *31*	*0.59*	*NS*	*0.01*	*201*	*NS*
	0.74 ± 0.84	− 0.07 ± 0.97	4.60	0.035*	0.06	134	0.036*

Dose 1, including LSD 200 μg plus ketanserin in Study 4 was used for pharmacokinetic statistics; Dose 2, excluding LSD 200 μg plus ketanserin condition in Study 4 was used for all LSD effect statistics; N, number of subjects; SD, standard deviation; AUC, area under the time-concentration curve; */**/***asterisks indicate level of statistical significance p < 0.05/0.01/0.001; F, F-value of the Analysis of variance; NS, not significant; ∆, values are change scores from placebo; η^2^, eta square; W, Wilcoxon signed-rank test statistic; ^a^p value of the Wilcoxon signed-rank test; *cursive text shows nominal values.*

Consistent with the pharmacokinetic effect of LSD (Fig. 1), CYP2D6 PMs exhibited a substantially longer duration of the acute subjective response to LSD (Table 1) and significantly greater alterations of mind compared with functional CYP2D6 subjects (Table 1). Specifically, ratings on the 5D-ASC total, AED subscale (including disembodiment, impaired control and cognition, and anxiety), and VR subscale (including complex and elementary imagery and changed meaning of percepts) significantly increased in PMs compared with functional CYP2D6 subjects (Table 1). CYP2D6 genotype had no relevant effect on the autonomic response to LSD (Table 1).

In contrast to CYP2D6, genetic polymorphisms of other CYPs, including CYP1A2, CYP2B6, CYP2C19, and CYP2C9, had no relevant effect on the pharmacokinetics or subjective or autonomic effects of LSD (Supplementary Tables S7 and S8).

## Discussion

The present study examined the influence of genetic polymorphisms on the pharmacokinetic and acute subjective effects of LSD in humans. The main finding was that genetic polymorphisms of CYP2D6 significantly influenced the pharmacokinetic and in part also the subjective effects of LSD.

LSD is metabolized almost completely in the human body. Only small amounts of the parent drug (~ 1%) are excreted in urine^39^. In vitro studies of human liver microsomes and human liver S9 fractions indicated a role for CYP enzymes in the metabolism of LSD^7,8^. CYP2D6 is involved in the *N*-demethylation of LSD to nor-LSD^8^. The present study provided additional in vivo evidence that CYP2D6 is involved in the metabolism of LSD in humans and that polymorphisms of the CYP2D6 gene influence both the metabolism of LSD and acute response to LSD in humans. Plasma nor-LSD concentrations in humans are mostly too low to be measured, even with highly sensitive methods^40^. However, we found an increase in both plasma LSD and O-H-LSD concentrations in individuals with a non-functional CYP2D6 genotype, consistent with the role of CYP2D6 in the formation of nor-LSD or other metabolites but not O-H-LSD. Thus, CYP2D6 is likely a crucial player in the degradation of LSD but not in the formation of its main metabolite O-H-LSD. The role of CYP2D6 could further be investigated in drug-drug interaction studies using LSD with and without selective CYP2D6 inhibition. This is also interesting because LSD may be therapeutically used in patients with psychiatric disorders and using a serotonin reuptake inhibitor (SSRI) treatment, which may also act as CYP2D6 inhibitors (e.g., fluoxetine and paroxetine)^41^. Consideration should also be given to discontinuing CYP2D6 inhibitors and allowing sufficient time for the enzyme to regenerate (up to 2 weeks) before LSD is used. Alternatively, in the presence of CYP2D6 inhibitors, the dose of LSD should be reduced, based on the present findings. On the other side, this might not particularly be the case for SSRIs. Chronic administration of antidepressants has been shown to decrease the number of 5-HT2 receptors in various brain regions due to receptor downregulation^42^. The slowly onset of 5-HT2A receptor downregulation together with the immediate inhibitory property of many SSRIs toward CYP2D6, could lead to an acute increase in LSD effects shortly after initiation of SSRI treatment but eventually to a decrease in effects as the primary target of LSD, 5-HT2A receptors, diminishe^43^.

With regard to other CYP enzymes, CYP2C19 was found to be involved in the formation of nor-LSD in vitro^7^. However, we found no influence of its genotype on the pharmacokinetics of LSD. Furthermore, CYP2C9 and CYP1A2 were reported to contribute to the hydroxylation of LSD to O-H-LSD^7,8^. CYP2C9 also catalyzes the *N*-deethylation to lysergic acid monoethylamide^7^. However, no effects of CYP2C9 genotype on the pharmacokinetics of LSD were observed in the present study in humans. For CYP1A2, no common loss-of-function polymorphisms have been identified to date. However, CYP1A2 is inducible by tobacco smoking in subjects with the common A/A genotype of the rs762551 SNP compared with the C/A and C/C genotypes^9^. Accordingly, we combined CYP1A2 activity inducibility with smoking status of the subjects (> 5 cigarettes per day = smoker). In a similar pharmacogenetic study with MDMA, we found higher 3,4-methylenedioxyamphetamine (MDA) levels (the minor metabolite of MDMA) in subjects who smoked 6–10 cigarettes daily and possessed the inducible genotype of CYP1A2 compared with subjects who smoked less and/or had the non-inducible polymorphism^15^. We did not find an influence of CYP1A2 genotype/smoking status on the pharmacokinetic of LSD in the present study. However, only five subjects were enrolled in the present study who met both requirements of being a smoker and possessing an inducible CYP1A2 genotype.

The pharmacogenetic influence of metabolizing enzymes on LSD appears quite similar to MDMA. For both psychoactive substances, LSD and MDMA, only polymorphisms of CYP2D6 appear to substantially impact pharmacokinetics and subjective effects^15^. However, because MDMA inhibits CYP2D6 and its own metabolism (i.e., autoinhibition), the effect of CYP2D6 genotype variations is limited and evident only during the onset of MDMA’s effects during the first 2 h after administration^16^. For LSD, moderation by CYP2D6 genotype appears to become more relevant later during the elimination phase, increasing the AUC and half-life of LSD and its duration of effect rather than its absorption and early effect peak. CYP2D6 PMs exhibited approximately 75% more total drug exposure than individuals with a functional CYP2D6 enzyme. We observed only a nonsignificant approximately 15% higher mean peak concentration. Therefore, total drug exposure, reflected by the AUC_∞_, was mainly determined by the lower elimination after the peak. This pattern was also present with the subjective effects of LSD. The VAS peak effects were not different between the different CYP genotypes, and the 5D-ASC ratings that reflected subjective alterations of mind over the entire day showed distinct differences that depended on CYP2D6 functionality. The non-functional CYP2D6 group reported an overall greater altered state of consciousness, with particularly higher ratings on the AED subscale, including Disembodiment, Impaired Control and Cognition, and Anxiety, and VR subscale, including Complex Imagery, Elementary Imagery, and Changed Meaning of Percepts.

Genetic effects on the acute subjective response to LSD is clinically relevant. Several studies in healthy subjects and patients found associations between the extent and quality of the acute subjective experience and long-term effects of psychedelics, including LSD^20–24^. Typically, greater substance-induced OB and more mystical-type effects could be associated with more favorable long-term effects. Specifically with regard to the 5D-ASC rating scale that was used in the present analysis, greater acutely psilocybin-induced OB and lower AED scores predicted better therapeutic outcomes at 5 weeks in patients with depression, whereas VR scores had no significant effects^20^. CYP2D6 PMs mainly had greater LSD-induced ratings of AED and VR but not OB, and these subjects may have an overall more challenging acute experience, with higher acute anxiety and possibly even lower therapeutic effects. This possible scenario needs further investigation. Geno- or phenotyping may be useful in patients who undergo LSD-assisted therapy. Based on the present findings, CYP2D6 PMs may benefit from approximately 50% lower doses than those that are used in functional CYP2D6 individuals. This possibility is also consistent with the observation that the higher LSD dose of 200 µg compared with 100 µg doubled plasma LSD exposure but did not result in higher ratings of OB but increased AED and anxiety on the 5D-ASC^17^.

The present study has limitations. Although this analysis was performed using the largest available sample of healthy human subjects who received LSD in placebo-controlled studies, the sample size is still relatively small. Although the sample size was sufficient to detect an effect of functionally very different genotypes (i.e., CYP2D6), it may have been too small to detect smaller changes with other CYPs. Additionally, CYP3A4 may play a role in the metabolism of LSD, but polymorphisms are rare^44^. Moreover, type I errors cannot be completely ruled out even if the hypothesis has been rationalized a priori. Drug-drug interaction studies with different selective CYP inducers/inhibitors are needed to confirm and expand the present findings.

The present study has several strengths, including the placebo-controlled design and use of validated psychometric tools. It also used statistical methods to address possible confounders. For example, complementary non-parametric analyses were used to confirm findings from parametric tests^45^. Additionally, the main analyses in this pooled study used z-transformed values to account for any between-study differences in genotype distribution and the doses used. Although this analysis is reliable for documenting changes between the tested genotypes, the measured values may only approximate the true size of the effect.

In conclusion, the present study revealed the influence of genetic polymorphisms of CYP2D6 on the pharmacokinetics and acute subjective effects of LSD in humans. Genetic polymorphisms of CYP2D6 significantly influenced the pharmacokinetic and subsequently subjective effects of LSD. No effect on the pharmacokinetics of LSD or response to LSD was observed with other CYPs. Given the potential therapeutic use of psychedelics, including LSD, the role of pharmacogenetic tests prior to LSD-assisted psychotherapy needs to be further investigated.

## Supplementary Information

Supplementary Information 1.

Supplementary Figure S1.

Supplementary Table S1.

Supplementary Table S2.

Supplementary Table S3.

Supplementary Table S4.

Supplementary Table S5.

Supplementary Table S6.

Supplementary Table S7.

Supplementary Table S8.
