# Supplementary material for: Genetic influence of CYP2D6 on pharmacokinetics and acute subjective effects of LSD in a pooled analysis
Source: Sci Rep. 2021 May 25;11:10851. doi: 10.1038/s41598-021-90343-y (PMC8149637; doi:10.1038/s41598-021-90343-y)
Supplement: Supplementary file 1 — Supplementary Information 1. [file 41598_2021_90343_MOESM1_ESM.docx]

**Supplementary Figure S1.** Linear regression model of body weight (kg) of the participants *vs*. plasma LSD exposure, expressed as AUC_∞_ (z-score). The LSD AUC_∞_ values were z-normalized per study. Dot colors indicate male (dark blue) or female (red) participants. Filled dot indicates a non-functional CYP2D6 genotype.
