# Supplementary figures and images for: Genetic influence of CYP2D6 on pharmacokinetics and acute subjective effects of LSD in a pooled analysis
Source: Sci Rep. 2021 May 25;11:10851. doi: 10.1038/s41598-021-90343-y (PMC8149637; doi:10.1038/s41598-021-90343-y)

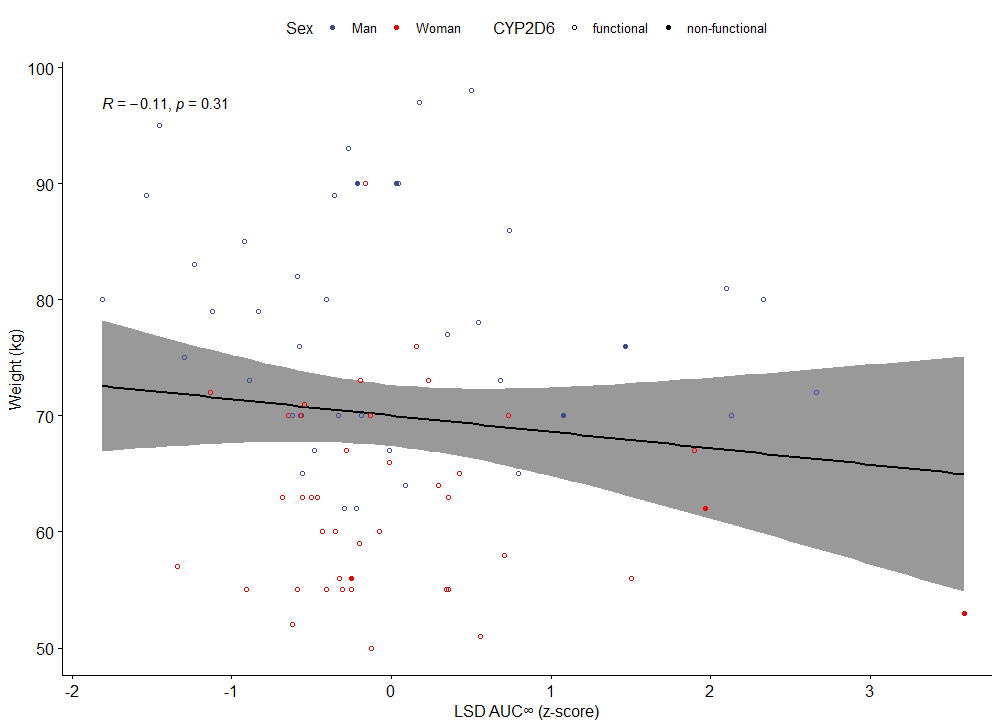

Supplement: Supplementary file 2 — Supplementary Figure S1. [file 41598_2021_90343_MOESM2_ESM.tiff]
